# Microfluidic-Assisted Growth of Perovskite Microwires for Room-Temperature All-Optical Switching Based on Total Internal Reflection

**DOI:** 10.1021/acs.nanolett.5c01866

**Published:** 2025-06-25

**Authors:** Annalisa Coriolano, Antonio Fieramosca, Laura Polimeno, Rosanna Mastria, Francesco Todisco, Milena De Giorgi, Luisa De Marco, Aurora Rizzo, Dario Ballarini, Ilenia Viola, Daniele Sanvitto

**Affiliations:** † 518742 CNR NANOTEC, Institute of Nanotechnology, via Monteroni, 73100 Lecce, Italy; ‡ CNR NANOTEC, Institute of Nanotechnology, c/o Dipt. di Fisica, Sapienza Università, P.le A. Moro 2, I-00185 Rome, Italy

**Keywords:** Perovskite, Exciton-Polariton, Waveguide, TIR, Gratings

## Abstract

The development of efficient integrated photonic circuits is fundamental for ongoing research in information processing and computer science. The greatest challenge facing photonic systems is achieving strong nonlinearities, which are exploitable in strongly coupled systems, leading to the formation of exciton-polaritons. In this context, the use of hybrid organic–inorganic perovskites (PVKs) offers a promising alternative, exhibiting robust interactions at Room Temperature (RT). However, the development of PVK-based integrated devices requires both the ability to achieve long in-plane propagation and the development of alternative fabrication approaches tailored to PVK materials, designed to preserve their optical properties and prevent degradation. Herein, we present the realization of a proof-of-concept all-optical switch using propagating polaritons confined in Total Internal Reflection (TIR), which ensures long in-plane propagation and limited optical losses. We realized efficient injection-extraction of the TIR-confined waveguide polariton modes by employing gold grating couplers prepatterned on the substrate.

## Graphical abstract

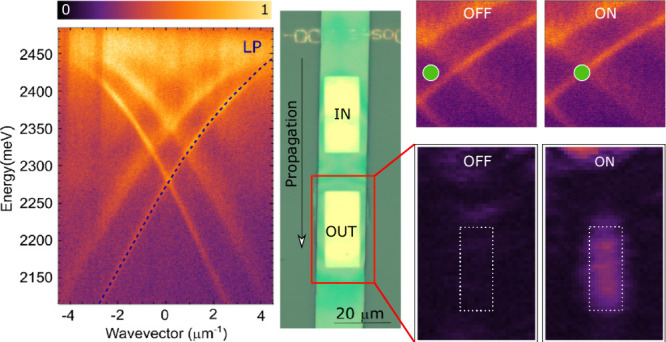

Exciton-polaritons, hybrid light-matter bosonic quasi-particles resulting from the strong coupling between excitons and photons, exhibit distinct characteristics inherited from their individual constituents.^1–3^ The photonic component contributes to a light effective mass (approximately 10^–5^ that of electrons), while the excitonic component introduces strong interparticle interactions (approximately 10^3^ times higher compared to standard nonlinear optical media).^4^ These peculiarities make them extremely sensitive to small changes in power, generally enabling long in-plane propagation, high switching efficiency, and no heat dissipation. Consequently, they fulfill the fundamental requirements for implementing all-optical computational schemes.^5–9^ From a more strictly technologically relevant perspective, exciton-polaritons can also be easily formed by employing high binding energy materials^10^ such as organics,^11–13^ transition metal dichalcogenides,^14–16^ and PVKs.^17–20^

In this regard, PVKs have attracted significant attention due to their strong exciton binding energy, high luminescence quantum yield, and narrow emission line width. More interestingly, these unique features can be tailored by modifying the crystals’ composition and shape through precise control of synthetic conditions.^21–24^

A plethora of fascinating results, such as polariton condensation, parametric scattering, and superfluidity, have been observed recently.^25–29^ It has also been demonstrated that polariton interactions in PVKs at RT can be as high as those in GaAs-based microcavities at cryogenic temperature, making PVKs highly attractive for polaritonic applications.^19^ The majority of these results have been obtained using a conventional planar microcavity with two distributed Bragg reflectors sandwiching the active layer. However, state-of-the-art PVK strongly coupled microcavities have high dissipation rates and short lifetimes as well as low group velocity, thus limiting the maximum achievable in-plane propagation and preventing applications in cascaded-on-chip technologies. Therefore, there is a high demand for the use of different optical platforms to fully harness the potential of PVK-based polaritons. In this regard, PVK single crystals, compared to their polycrystalline counterparts, exhibit superior properties owing to their reduced defect density, which enhances the exciton lifetime and diffusion, making them an ideal platform for photonic application. Alternatively to using planar microcavities, PVK single crystals can support the formation of exciton-polaritons without the need for external resonators,^18,30,31^ due to the high refractive index contrast between them and the external environment. They can efficiently support propagating modes, such as waveguide modes, where optical confinement is achieved through TIR, making them highly suitable for the realization of active polaritonic circuits. The primary benefit of this system lies in the ability to achieve significant in-plane wavevectors, resulting in high group velocities that greatly exceed those of planar microcavities with an equivalent lifetime. Moreover, this configuration requires less time for fabrication, minimizes the mode volume, and reduces the optical losses.

The significant interest in this direction is evidenced by the recent development of PVK-based waveguide couplers, interferometers, and beam splitters.^18,32–34^ To achieve this, the shape and quality of the crystals must be appropriately tuned to obtain sharp edges, uniform surfaces, and geometric parameters that support TIR-propagating modes and minimize optical losses. A key factor in obtaining single crystals with the desired shape and features is the confinement of the precursor solution. Among various synthetic approaches, microfluidic assisted confined crystal growth has emerged as an effective method for producing high-quality PVK single crystals of a desired shape.^17,18,34,35^

However, although PVK-based optical circuitry represents a highly promising photonic platform, the crystals developed until now lack efficient injection/extraction since the TIR-propagating signal is collected from the edges. A robust design necessitates efficient light coupling achieved through a grating coupler, which typically involves postprocessing steps utilizing polar solvents that may impact the quality of the active material.^36,37^

In this work, we overcome these limitations by leveraging the microfluidic-assisted growth technique to directly fabricate a 2D PVK waveguide on a gold grating. This technique enables precise control over the dimensions, shape, and position of the active material on the final device, thereby removing constraints associated with fabricating an optical grating using post-processing fabrication techniques.^38,39^

The effectiveness of this method is demonstrated by the synthesis of a high-quality dodecylammonium lead halide PVK microwires, (C12)_2_PbI_4_ (C12 = dodecylammonium, C_12_H_25_NH_3_^+^), consisting of alternating layers of a PbI_6_^4–^ octahedra layer interspaced with a double layer of C12. The long alkyl chain organic spacer enhances the environmental stability of the crystal while also ensuring a high exciton binding energy.^40–42^ Employing a single grating configuration, we demonstrate how waveguide polaritons, off-resonantly excited, can propagate over considerable distances into the PVK microwire and be efficiently extracted by the gold grating placed far from the excitation spot. Moreover, by exploiting the exciton-polariton nonlinearities and a coherent resonant excitation in a double grating configuration, we realize a proof-of-concept polariton switch operating in TIR. Our approach demonstrates a fully integrable, robust, and reproducible polariton device, showcasing significant potential for practical applications in advanced photonic circuits and optoelectronic systems.

The sample structure consists of C12-PVK microwires grown on a substrate with gold gratings fabricated on commercial glass. The chemical structure of the C12-PVK is depicted in Figure 1a. The inorganic layers of PbI_6_^4−^ octahedra are intercalated by long alkyl chains, resulting in a separation of 24.437 Å between the two nearest least-squares planes passing through the equatorial atoms of the PbI_6_^4−^ octahedra.^43^ The presence of a long ammonium chain can improve device stability under environmental conditions.^44^ Initially, a layer of poly­(methyl methacrylate) (PMMA) is spin-coated onto the glass substrate, followed by electron-beam lithography of the grating and subsequent development. Then, 60 nm of gold is thermally evaporated, followed by a lift-off process in acetone (see Section 1 in the Supporting Information for further details). Using a home-built micromanipulator (Figure S1), a patterned polydimethylsiloxane (PDMS) replica with microchannels having a height of 500 nm and variable widths is aligned with the gold gratings (Figure 1b), and the substrate was brought in conformal contact with the PDMS replica. C12-PVK microwires are prepared by using a microfluidic-assisted technique from a precursor solution, which is deposited at one end of the microchannels (Figure 1c). Capillary forces guide the solution into the microchannels, filling them and ensuring the formation of C12 microwires through confined nucleation. The growth process is fine-tuned by optimizing key parameters, such as temperature, precursor solution concentration, solvent type, and ambient solvent saturation. This optimization is essential for obtaining sharp edges and low roughness, reducing scattering losses, and enabling long-range polariton propagation. To achieve high crystal quality, a stoichiometric solution of precursors in γ-butyrolactone has been used, with the growth process conducted at RT. During the nucleation phase, the substrate with the template filled with the precursor solution is placed in a sealed box. This setup creates a solvent-saturated environment that slows the nucleation phase, promoting the formation of a low number of nuclei. These nuclei evolve into PVK microwires as the solvent of the precursor solution gradually evaporates (Figure 1d). After 8 h, the growth is complete, and the PDMS replica is removed. Millimeter-long PVK microwires are formed, positioned on the substrate atop the gold gratings (Figure 1e). Scanning electron microscope images of the as-synthesized microwires highlight the high quality of C12 crystals (Figures S2 and S3).

**Figure 1:**
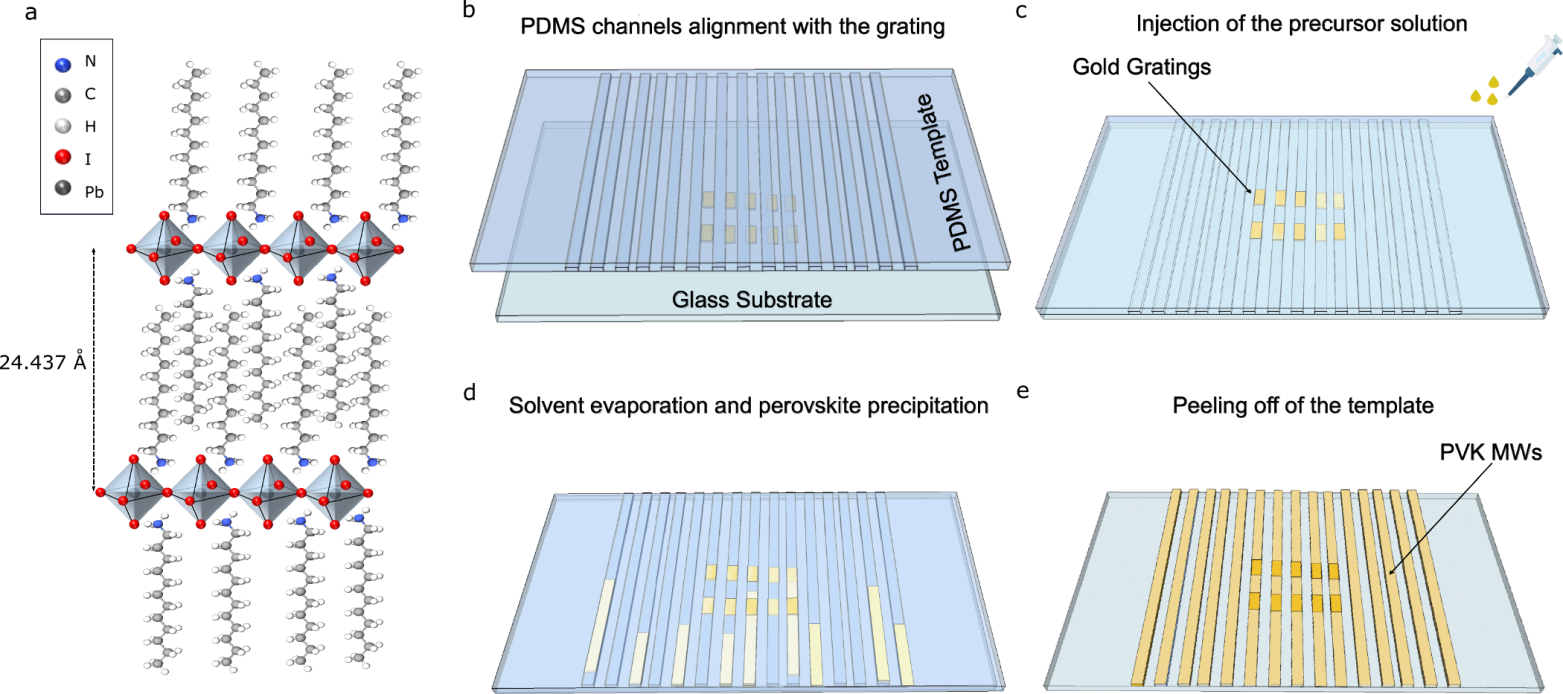
(a) 3D schematic of the C12-PVK structure. Illustration of the template-confined growth process for C12 microwires. First, the patterned PDMS template is aligned with the gold gratings (b) and transferred onto the glass substrate. The precursor solution is then injected into the PDMS microchannels (c), initiating solvent evaporation and PVK precipitation (d). After 8 h, the solvent is fully evaporated, resulting in the formation of microwires. Once the template is peeled off, single crystal microwires remain on the glass substrate (e).

The periodicity of the grating is designed to allow for the extraction of the signal confined in TIR within the microwires. Rigorous coupled wave analysis^45^ is employed to simulate the optical response of the grating, considering a C12-PVK microwire with a thickness of 500 nm grown on top. The real and imaginary parts of the PVK refractive index are taken from ref 46. The theoretical energy versus in-plane momentum (*k*_∥_) reflectivity map, considering Transverse Electric (TE) polarized light, is reported in Figure 2a. The formation of strongly coupled propagating and counterpropagating lower polariton (LP) waveguide modes is clearly visible. The characteristic dispersion of the waveguide modes, which is linear in the weak coupling regime, exhibits a notable increase in curvature as the energy nears the exciton resonance (*E*_exc_ = 2525 meV, indicated by the white dashed line in Figure S4, Section 4), thus providing clear evidence of the achievement of strong coupling.

**Figure 2:**
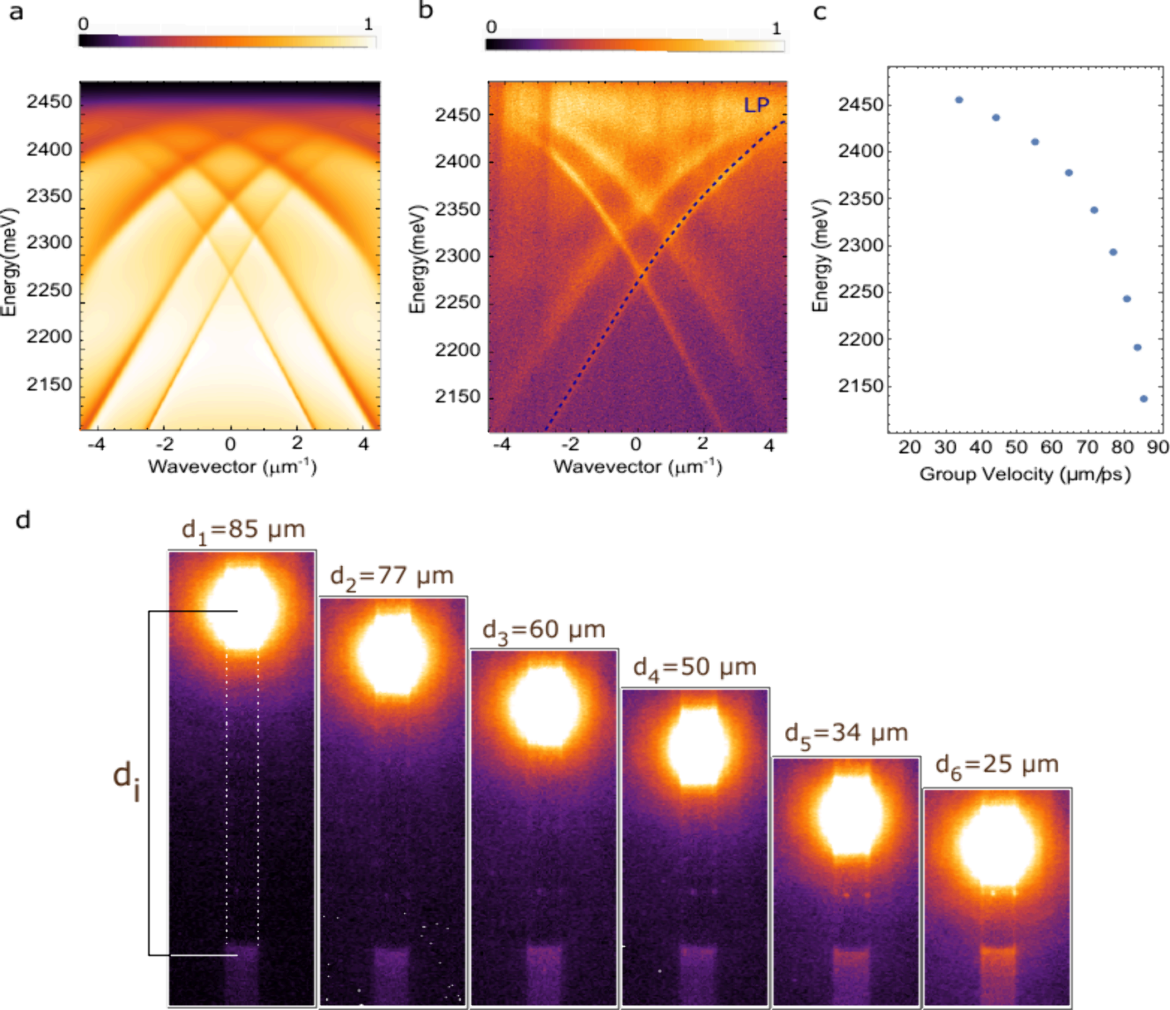
(a) Theoretical energy versus in-plane momentum reflectivity map, considering TE polarized light. (b) Experimental energy versus in-plane momentum reflectivity map, considering TE polarized emitted light. The extracted Rabi splitting is 340 meV. The dashed blue line indicates the dispersion of one of the manifold LP waveguide modes. (c) Group velocity as a function of energy for the mode marked by the dark blue dashed line. (d) Real-space PL maps as a function of the distance *d*_*i*_ between the excitation spot and the outcoupler grating.

The formation of the upper polariton (UP) branch can be seen in Figure S4, where the simulation is performed in a broader energy region. The appearance of multiple LP waveguide modes is a direct consequence of the free spectral range, which, with 500 nm of PVK, supports the formation of multiple waveguide modes. Moreover, the dispersion of the bare grating in the same spectral region and TE polarization is shown in Figure S5 and does not display the formation of any lattice mode, thus confirming that the adopted design effectively works as an outcoupler for the TIR-confined waveguide polariton modes. Experimentally, the grating is directly tested using momentum resolved photoluminescence (PL) measurements. The grating region is excited in the center by using a continuous wave (CW) off-resonant pumping (λ = 405 nm) and the PL signal collected in TE polarization. The resulting energy versus in-plane momentum (*k*_∥_) PL map is shown in Figure 2b and is in good agreement with the theoretical calculation. It is important to note that the visibility of the UP is hindered by the strong absorption at high energy, as previously reported.^30,43,47,48^ From the fit of the experimental data (blue dashed line in Figure 2b), we obtained a Rabi splitting of Ω_TE_ = 340 meV, which is in good agreement with previous reports.^18,49,50^ Moreover, with reference to the mode highlighted in Figure 2b, we calculated the group velocity of the mode as a function of energy, *v*_*g*_(*E*), evaluated as follows: *v*_g_ = 1/ℏ × ∂E/∂k. Depending on the excitonic fraction, the group velocity ranges from 30 to 90 μm/ps, that is 1 order of magnitude higher than that of standard microcavities^7^ and comparable to that of propagating surface modes, such as Bloch Surface Waves (BSW).^51,52^

As discussed above, waveguide modes are capable of harnessing propagation properties. However, it is crucial for PVK microwires to exhibit minimal scattering to preserve the benefits of working with TIR-confined optical modes. To assess the quality of the PVK microwires, we employed a single grating configuration, wherein the material is off-resonantly excited at various distances (*d*_*i*_) from the outcoupler grating, as reported in Figure 2d. From the real space PL maps, we have estimated the emission intensity collected below the outcoupling region for each distance, $I_{d_{i}}^{Out}$ . With this approach we evaluate the propagation loss coefficient α = 0.12 dB/μm, as reported in Figure S6, Section 5. This low value is a direct consequence of the microfluidic-assisted growth method employed here, which significantly reduces impurities and ensures sharp edges and long-range homogeneity. Compared to similar wires, such as CsPbBr_3_ wires grown via the capillary bridge method and integrated into planar microcavities,^7^ it appears slightly lower, thereby confirming how the additional deposition steps affect the optical losses.

Although the PL measurements discussed above clearly confirm the excellent optical quality of the microwires, the realization of on-chip integrated devices and circuits relies on efficient injection and extraction of a coherent propagating signal. Recently, various optical structures such as waveguide couplers, interferometers, beam splitters, X-couplers, and Y-couplers have been theoretically investigated and experimentally realized in PVK films using techniques such as Focused Ion Beam lithography or assembling PVK quantum dots with a capillary bridge technique.^32,33,53^ In these studies, coherent propagation is achieved through optical pumping of the active material above the lasing threshold. However, these implementations lack efficient injection/extraction, as the structures are pumped off-resonantly and the TIR-propagating signal is collected from the edges. Here, we implement an ideal scheme in which a resonant injection and the extraction of the TIR-propagating signal are performed with two gratings.

The PVK microwires are grown on top of two identical gratings, spaced approximately 10 μm from each other (Figure 3a). The top grating is utilized to perform a resonant injection of a CW laser with a photon energy of 2330 meV and a well-defined in-plane momentum, *k*_∥_ = 1.12 μm^–1^, resulting in a group velocity of *v*_g_ = 62.27 μm ps^–1^ and a coupling efficiency ranging from 9% to 15% (see Figure S7). The real space map is shown in Figure 3b, while the pumped area is indicated by the green circle in the zoomed-in energy versus *k*_∥_ map displayed in Figure S7a. By carefully matching the in-plane momentum of the waveguide mode, it is possible to obtain a propagating polariton flow with a well-defined velocity that is out-coupled from the second grating.

**Figure 3:**
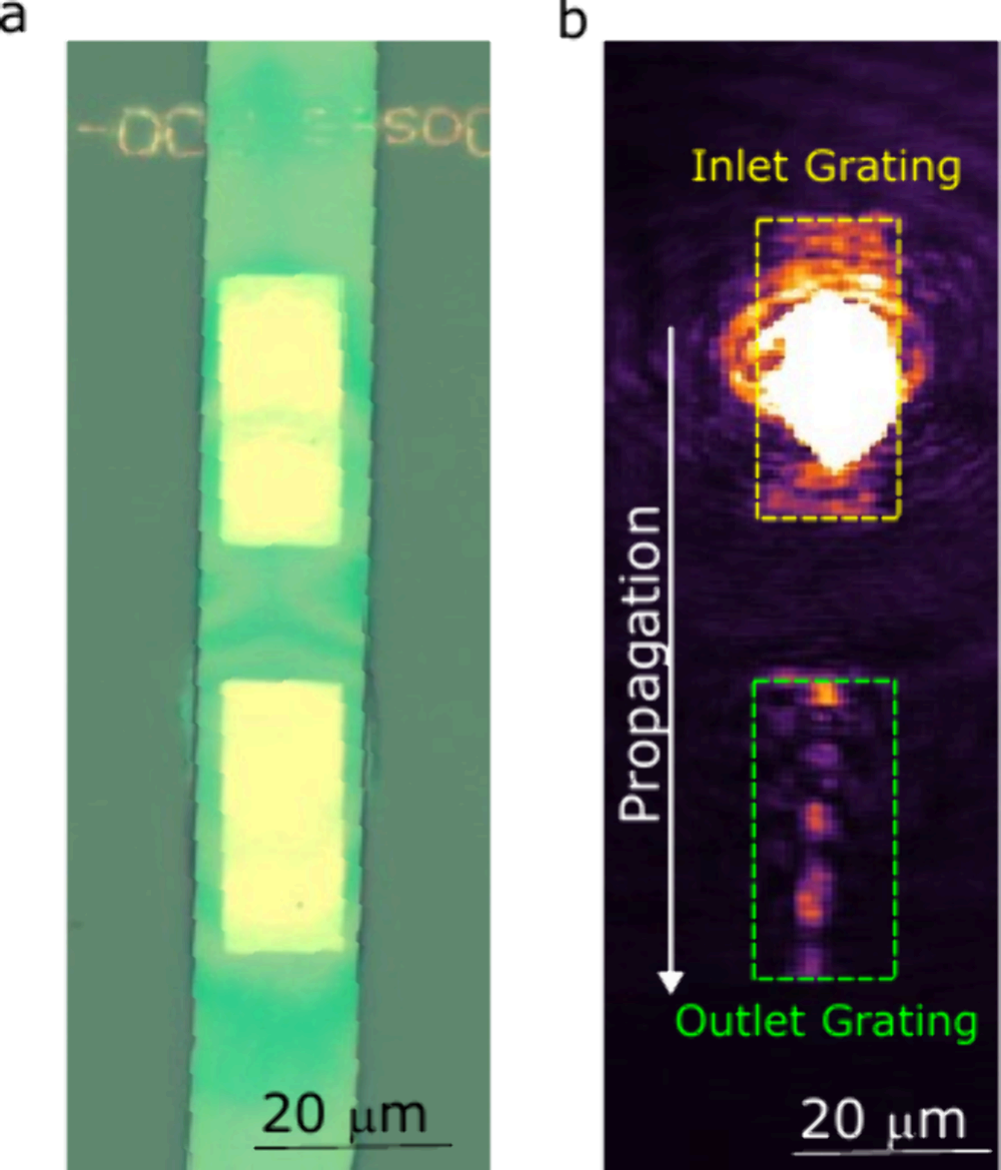
(a) Optical image of C12 microwire grown on the top of gold gratings. (b) Real-space resonant propagation. The excitation spot is placed on the top inlet grating (dashed yellow rectangle) while the bottom grating (dashed green rectangle) outcouples the radiation. The white arrow indicates the propagation direction.

It is important to note that the outcoupled signal shows an interference pattern on the grating region. This is a direct consequence of the laser coherence, which, once it reaches the outcoupler region, backscatters at the opposite in-plane momentum, thereby creating the interference pattern visible in real space. Note that such an interference pattern demonstrates the long propagation even under the diffraction grating.

This is in contrast with the real space PL maps shown in Figure 2d, which instead display a uniform signal that spreads over the whole grating region.

Based on the results shown above, we experimentally demonstrate that our samples exhibit a robust design characterized by long in-plane propagation and high velocities. Consequently, it is natural to explore experimental configurations capable of realizing logic operations, thereby harnessing polariton nonlinearities. Within the exciton-polariton framework, various proof-of-concept logic elements have been successfully implemented in GaAs-based planar microcavities.^5,54^ However, cost-effective and technologically relevant polariton devices require RT operation, presenting a significant challenge for conventional inorganic semiconductors due to their low exciton binding energy. Only a few polariton devices operating at RT have been demonstrated so far. While organic microcavities and transition metal dichalcogenides have enabled all-optical polariton transistor and switches, they do not use in-plane propagation.^11,55,56^ Conversely, recent advances include a nonlinear polariton source using propagating BSWs in strongly coupled WS_2_ monolayers,^51^ both exploiting propagation properties. In this context, our platform offers an optimal trade-off between minimizing footprint and harnessing in-plane propagation. To this end, we initially investigated the existence of a nonlinear response under optical pumping in our samples (Figure S8, Section 6). One of the two gratings is resonantly pumped using a pulsed laser (2250 meV, 10 kHz, 100 fs) in a reflection configuration. The laser is kept broad in momentum space, and the LP waveguide modes are discernible as dips in the laser’s spectrum, as visible in the energy vs *k*_∥_ reflectivity map collected at low excitation power and reported in Figure S8b. By increasing the pumping power, a clear blue shift becomes evident, as shown in Figure S8c. This observation aligns well with previous findings. It is important to note that the total energy shift exceeds the line width of the LP waveguide modes (see Figure S8d,e), therefore satisfying the necessary conditions for implementing a polariton-based all-optical switch. To fully exploit the proven strong nonlinearities in a real device, we present an experimental demonstration of this proof-of-concept all-optical switch. For this specific experiment, we employed two lasers: a fs pulsed laser, which shifts the energy position of the LP waveguide modes, and a CW laser, acting as the propagating signal within the microwire. The details of the measurement configuration are reported in the SI. A scheme of the configuration is depicted in Figure 4a. Then, we evaluated the intensity emitted below the outcoupler grating (dark green square in Figure 4a) and compared it with the intensity immediately before (dark blue square in Figure 4a), i.e., the residual scattered signal not associated with the propagating CW beam. The polariton dispersion relations for the OFF and ON states are presented in Figure 4b,c, respectively. The observed blue shift in the ON state highlights the nonlinear effects arising from polariton–polariton interactions. We performed this analysis as a function of the pumping power of the pulsed laser, as reported in Figure 4d for regions 1 (dark green points) and 2 (dark blue points), respectively. Under these experimental conditions and at low pumping power, the system is in the OFF-state regime. This means that the signal collected below the outcoupler grating, associated with the propagating CW laser, is negligible, as very little light is coupled into the propagating mode. This low-power regime is evidenced in Figure 4d by the red band on the left side of the figure. In contrast, as the pump power is increased, the LP waveguide modes undergo a blue shift, and the CW laser becomes resonant with the propagating mode. Consequently, polaritons are injected below the incoupler grating, propagate and are detected below the outcoupler grating; i.e., the system reaches the ON-state regime. This high-power regime is depicted in Figure 4d by the yellow band on the right side of the figure. The nonlinearity results in a clear “kink” in the signal detected below the grating, distinctly different from the scattered signal. This two-beam experiment effectively proves how the designed sample can act as an all-optical polariton switch and demonstrates the realization of an active polariton waveguide. Based on these measurements, we obtained a switching contrast between 8 and 11 dB, indicating a clear and robust switching behavior, with a switching threshold in the range of 30–60 μJ/cm^2^. Details on the relation used to evaluate these parameters and a comparison with the state of the art are provided in Section 7 of the SI. Moreover, we discuss strategies to reduce propagation losses and improve grating efficiency in Section 8.

**Figure 4:**
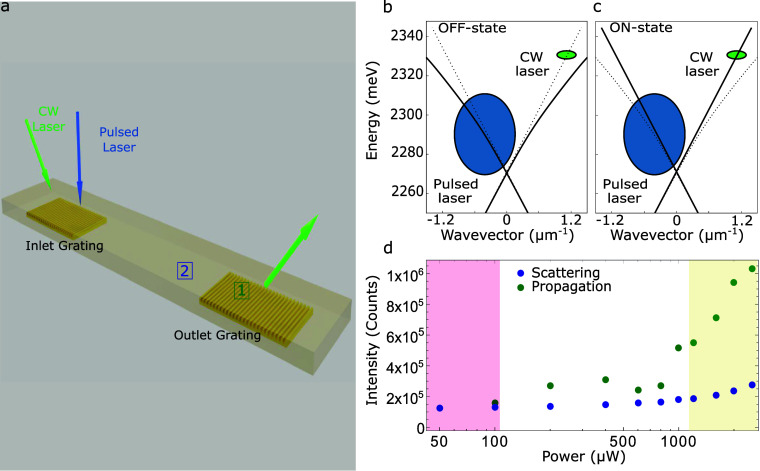
(a) Sketch illustrating the optical measurement configuration where a fs pulsed laser (blue arrow) allows the gating of a CW laser (green arrow), therefore acting as an ON-OFF switch of the propagating signal within the microwire. (b,c) Polariton dispersions in the OFF (b) and ON (c) states. Blue circles denote the *k*-position of the pulsed excitation, while green circles indicate the k-position of the CW laser. (d) Power-dependence of the intensity (counts) of the propagating signal collected in region “1” (dark green dots) and the scattering signal (dark blue dots) collected in region “2” of the microwire. The red and yellow bands indicate the OFF and ON state regime, respectively.

In conclusion, we demonstrated an active polariton waveguide based on propagating polaritons sustained by a dodecylammonium lead halide PVK microwire. A microfluidic-assisted growth technique is employed to fabricate waveguide-based devices, allowing control over the material’s dimensions, shape, and position. We achieved efficient injection and extraction of TIR-propagating polaritons by growing the 2D PVK microwire directly on a gold grating. Leveraging the excellent propagation and strong polariton-polariton interactions in these PVK waveguides, we realized an all-optical switch operating at RT. The method yields a robust, reproducible device with minimal footprint and no postprocessing steps that might degrade material quality.

We emphasize that PVKs offer high nonlinearity, good processability, and efficient in-plane propagation, unlike transition metal dichalcogenides and organics, which suffer from shorter propagation and lower nonlinearities. This makes PVKs strong candidates for scalable polaritonic devices.

## Supplementary Material



## Notes

Additional information about device fabrication, microwire synthesis, optical measurements, simulation and fits, estimation of the propagation losses, grating efficiency and polariton nonlinearities, key figures of merit of the switch, and propagation loss mechanisms and strategies to improve coupling efficiency (PDF)
