# Supplementary material for: Microfluidic-Assisted Growth of Perovskite Microwires for Room-Temperature All-Optical Switching Based on Total Internal Reflection
Source: Nano Lett. 2025 Jun 25;25(27):10794–801. doi: 10.1021/acs.nanolett.5c01866 (PMC12257638; doi:10.1021/acs.nanolett.5c01866)
Supplement: Supplementary file 1 [file nl5c01866_si_001.pdf]

Supporting Information: Microfluidic-Assisted Growth of Perovskite Microwires for Room-Temperature All-Optical Switching Based on Total Internal Reflection

Annalisa Coriolano[1†], Antonio Fieramosca[1†*], Laura Polimeno[1], Rosanna Mastria[1*], Francesco Todisco[1], Milena De Giorgi[1], Luisa De Marco[1], Aurora Rizzo,[1] Dario Ballarini[1], Ilenia Viola[1,2], Daniele Sanvitto[1]

Section 1: Microfluidic-confined growth

PDMS template fabrication

The patterned PDMS template was obtained starting from a silicon master (ThunderNIL) having an array of microchannels with a height (h) of 500 nm and a width (w) ranging from 1 to 20 μm. A micro-structured PDMS template, featuring channels with dimensions h: 500 nm and w: 2-20 μm, is created by blending the prepolymer and curing agent in a 10:1 weight ratio. The mixture is then placed in a desiccator for 30 minutes to eliminate any bubbles. Subsequently, the liquid PDMS is cast onto a commercially patterned silicon master (ThunderNIL). After meticulous removal of air bubbles, the PDMS is cured in an oven at 140°C for 15 minutes. The elastomeric replica was then detached from the master and placed in conformal contact with the device substrate, with the microchannels open to facilitate the microfluidic injection of a small volume of precursor solution.

C12 MWs growth

n-dodecylammonium iodide, Acetone, Isopropanol (IPA), are purchased from Sigma Aldrich, Polydimethylsiloxane (PDMS) Sylgard 184 is purchased from Dow-Corning. Lead(II) iodide (PbI_2) is purchased from Alfa Aesar. γ-butyrolactone (GBL) is purchased from TCI. All chemicals are used as received without any further purification. The precursor solution is prepared within a N_2-filled glovebox by dissolving n-dodecylammonium iodide (156.63 mg, 0.5 mmol) and lead iodide (115.25 mg, 0.25 mmol) in 1 mL of γ-butyrolactone. This process yielded a 0.25 M solution, which is stirred at 70 °C for 1 hour until achieving a clear yellow solution.

The alignment of the patterned template with the gold gratings on the glass substrate is achieved using a custom-built micromanipulator in coordination with the PDMS channels. A schematic representation of the setup is depicted in Figure S1. The xy micrometer stage is equipped with a rotator to facilitate the alignment between the PDMS channels and the gold gratings.

Once aligned the PDMS template on top of the gold gratings, the growth process is initiated by placing 1 μL of the precursor solution at one end of the PDMS microchannels, by using a micropipette. Capillary forces guide the solution, allowing it to fill the channels of the PDMS template seamlessly. The flow within the microchannels in similar condition remains constant, as it is primarily determined by the geometric parameters of the channel, such as length, width, and height, as well as by the viscosity of the precursor solution. The latter is influenced by the type of solvent and the precursor concentration used. Once optimized, maintaining all these parameters constant ensures consistent growth, yielding microwires with similar morphology and crystal quality across different syntheses.

The growth is carried out in a box sealed with parafilm and left at room temperature. As the solvent gradually evaporates, crystal precipitation occurs within the channels of the template. After 8 hours, the PDMS template is carefully removed, resulting in high-quality C12 microwires perfectly aligned with the gold gratings on the glass substrate.

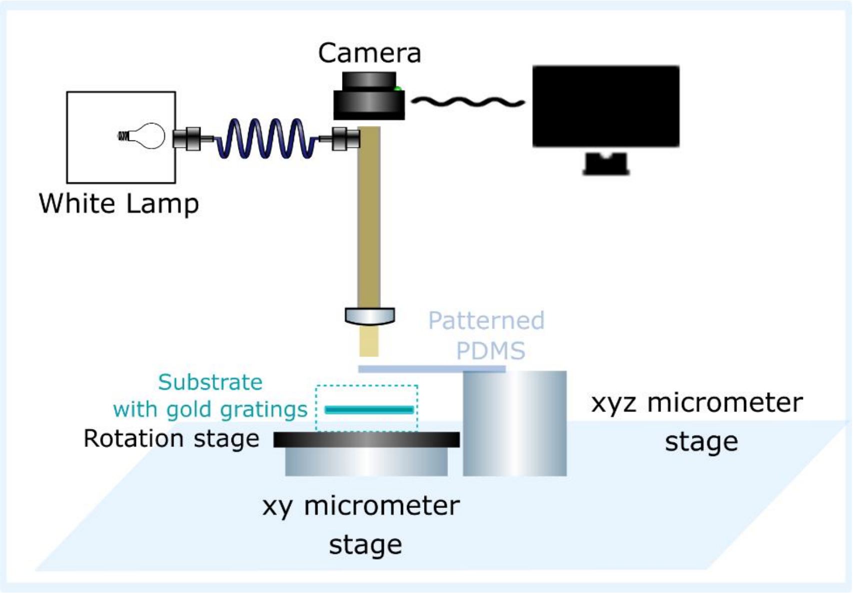

Figure S1. Sketch of the home-built micromanipulator used to align the PDMS replica with the gold gratings.

This approach allows for the fabrication of microwires with uniform height, defined by the PDMS template, and lengths higher than 200 μm (see Figure S2). In this work, we tested channel widths ranging from 2 μm to 20 μm to demonstrate the versatility of the technique in obtaining crystals with desired shapes. It is worth noting that, under identical solvent types and precursors concentration, the solution flows more easily in wider channels, often resulting in longer microwires. However, by opportunely adjusting the precursor solution, it is possible to grow very long microwires even in narrow channels, such as those with a width of 2 μm. The width of the channel influences also the solvent evaporation rate, with wider channels leading to faster evaporation.

We explored different strategies to control the growth rate and to achieve an optimal growth velocity that ensures good morphology and crystal quality. Among the tested parameters, such as temperature and solvent saturation, we found that controlling solvent saturation in the growth environment is the most effective for finely tuning the process. Both sealed systems and the use of a solvent reservoir have proven to be effective in slowing down crystallization, even under room temperature conditions.

Figure S2 and Figure S3 show SEM images of the C12 PVK MWs single crystals obtained using the microfluidic-assisted approach for growth on a glass substrate. In particular, we used a template with multiple channels of different lateral dimensions. The image reveals a flat and uniform surface with sharp edges, free from defects.

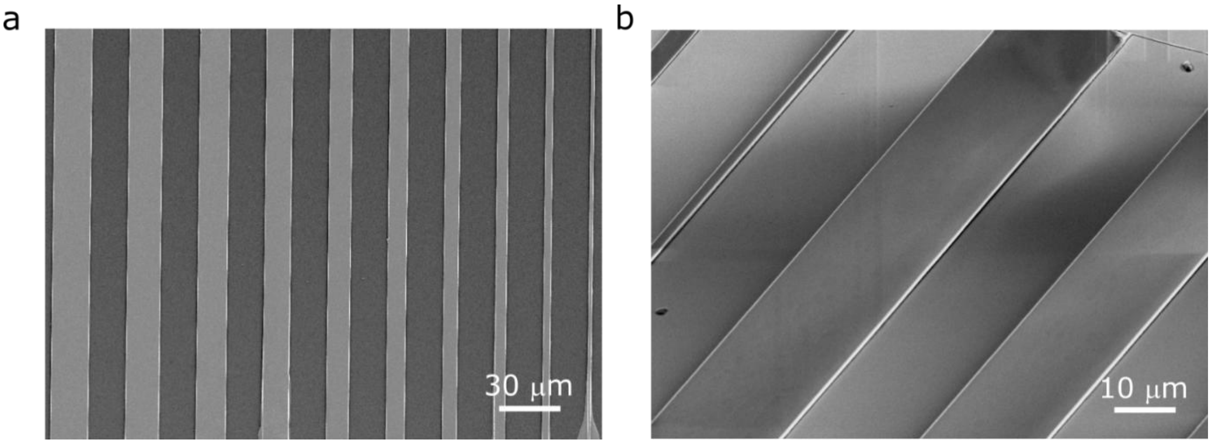

Figure S2. SEM images of the C12 PVK MWs on glass substrate.

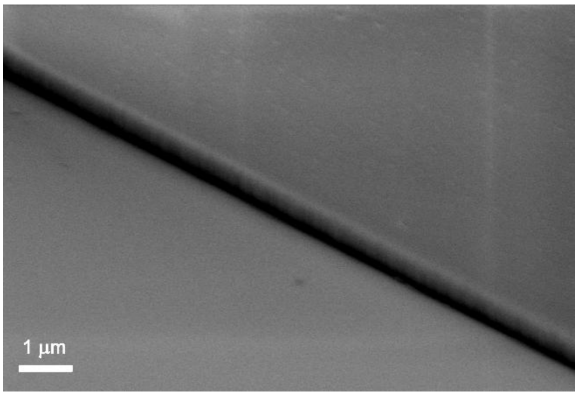

Figure S3. High-magnification SEM image highlighting the well-defined edge of a C12 microwire crystal.

Section 2: Gratings fabrication

Glass substrates are cleaned thoroughly with acetone and IPA in an ultrasonic bath, followed by a dehydration bake of 10 minutes at 180°C on a hotplate. Polymethyl methacrylate (PMMA) is used as an electron beam resist (MicroResist, 950 PMMA A4) and is spin-coated on cleaned samples at 3000 rpm for 60 seconds. Subsequently, the samples are annealed at 180°C for 180 seconds. Finally, in order to avoid surface charging during electron beam lithography, a conductive polymer is spin coated on the samples (MicroResist, DisCharge H_2O) at 2000 rpm for 60 seconds, followed by a soft baking at 90°C for 60 seconds.

E-beam lithography is performed on a 30 kV column system (Raith). Written samples are then immersed in water to remove the thin conductive polymer and then finally developed in a methyl isobutyl ketone:IPA 1:3 solution for 60 seconds. Finally, 3 nm of chromium and 60 nm of gold are thermally evaporated onto the substrates, followed by overnight lift-off in acetone at room temperature.

Section 3: Optical Measurements

All the optical measurements are performed in ambient conditions at RT. For the photoluminescence measurements, the microwire is off-resonant excited by using a CW diode laser ($\lambda = 405$nm). The photoluminescence is collected in reflection configuration, using a long-working distance objective (Olympus, 40x/NA=0.6). The angle resolved dispersion is taken by imaging the back focal plane of the objective onto the entrance slits of a spectrometer (Princeton Instruments, Acton Spectra Pro SP-2300) equipped with three gratings (150 lines/mm, 300 lines/mm, 600 lines/mm) and coupled to a 2D charge-coupled device array (Princeton Instruments, Pixis 400).

For the all-optical switching measurements, two sources are employed: a tunable femtosecond laser and a CW diode laser operating at 532 nm. The pulsed source is derived from a Ti:sapphire laser (Coherent, Vitara) that is pumping an ultrafast amplifier (Coherent, Legend). The amplifier is coupled with a computer-controlled Optical Parametric Amplifier for wavelength modulation (Coherent, TOPAS). The repetition rate and pulse width are 10 kHz and 100 fs respectively. The CW laser is focused at a given in-plane momentum and slightly off-resonance with respect to the LP waveguide mode. The off-resonance condition is achieved by precisely tuning the CW laser to an in-plane momentum slightly smaller than that of the LP waveguide mode at the same energy ($k_{//} = 1.12\ \mu m^{-1}$). In real space, the Full Width at Half Maximum (FWHM) of the CW laser spot is approximately 3 μm. The pulsed laser, with a linewidth of about 40 meV and centered around $E_{\text{pulsed}} = 2290$ meV, is on-resonance with the LP waveguide mode. To prevent any undesired propagation of residual signals from the pulsed laser at the out-coupler grating, it is configured to excite the LP waveguide mode propagating in the direction opposite to that of the CW laser. This experimental configuration allows us to exclusively monitor the modifications induced by the pulsed beam on the propagating CW laser.

It is important to note that the pulsed laser is responsible for opening temporal windows that allow the propagation of the CW laser at 10 kHz. Although the temporal width of these windows also depends on the polariton lifetime and the microscopic mechanisms driving the nonlinear response[1], the repetition rate is relatively low, and the switching effect can only be observed in time-integrated measurements, averaged over several pulses, in order to accumulate sufficient propagating counts on the output grating. For this reason, the data presented in Fig. 4 are integrated over 20 seconds for each pumping power. Additionally, the choice of the initial state is crucial. In our experiments, we always performed switching-ON measurements, meaning that at low pumping power, no counts are associated with the propagating signal on the output grating. This is advantageous for the measurements, as it ensures a clear distinction between the signal at low and high pumping powers.

Section 4: Simulation and fits

The dispersion patterns presented in Figure 2a are modeled using the semi-analytical Rigorous Coupled-Wave Analysis (RCWA) method as implemented by the S4 package.

The experimental data depicted in Figure 2b are fitted utilizing a 2x2 coupled harmonic oscillator Hamiltonian:

$$\begin{pmatrix} E_{ph}(k) & \Omega/2 \\ \Omega/2 & \mathrm{E}_{exc} \end{pmatrix}$$

Here, E_{exc} represents the exciton energy, Ω denotes the Rabi splitting, and $E_{ph}(k)$ is the energy dispersion of the photonic branch.

Figure S4 presents the theoretical reflectivity map, for TE polarization, calculated using the same parameters as those used for the map shown in Figure 2 of the main text. This map illustrates the relationship between energy and in-plane momentum (*k*) over a broader energy range, enabling a clearer visualization of the upper polariton branches, otherwise hidden by the high absorption of perovskite in the experimental measurement.

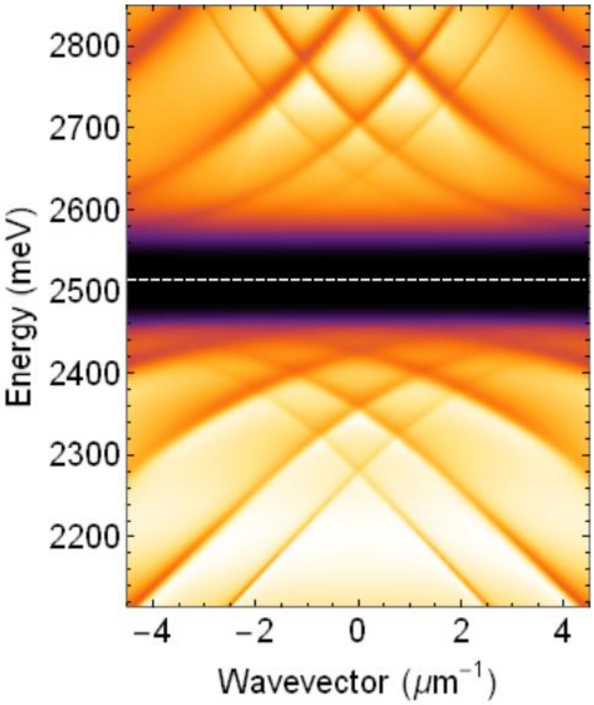

Figure S4. Simulated reflectivity map showing Energy versus In-plane momentum (*k*), performed over a broader energy range to reveal the upper polariton branches. The white dashed line indicates the exciton position (E_{exc} = 2525 meV).

Figure S5 shows the simulated reflectivity map of Energy versus In-plane momentum, *k*, for the bare grating in the same spectral region as the dispersions reported in Figure S4, for TE polarization. This map does not display the formation of any lattice modes, thus confirming that the adopted design effectively functions as an out-coupler for the TIR-confined waveguide polariton modes.

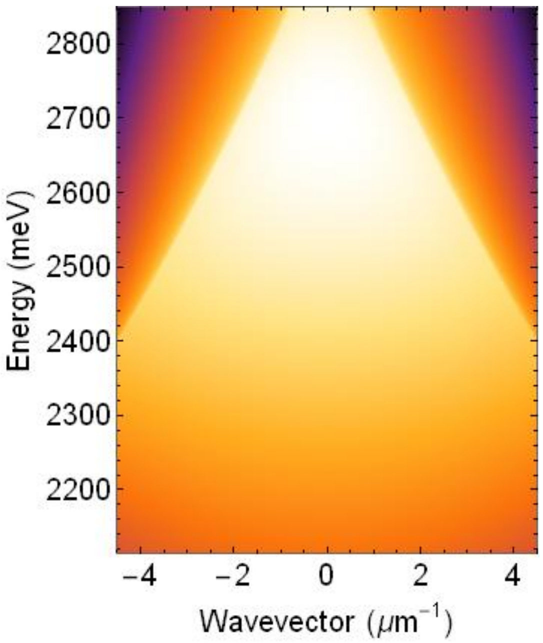

Figure S5. Theoretical reflectivity map of Energy versus in-plane momentum *k* of the bare 1-D gold grating on glass substrate.

Section 5: Estimation of propagation losses

From the real space PL maps, we have estimated the emission intensity collected below the outcoupling region for each distance, $I_{d_i}^{Out}$. With this approach we evaluate the propagation loss coefficient α, following the relation: α = (1/L) 10 log ($I_{d_i}^{Out}$/$I_{d_0}^{Out}$), where $I_{d_i}^{Out}$ is the intensity at every distance, $I_{d_0}^{Out}$ is the intensity at the shortest distance, and $\Delta L = (d_i - d_0)$ the relative distance, with d_0 taken as the reference point for the shortest distance. Therefore, we have extracted a loss coefficient α for each pair of measurements, as reported in Figure S6 and obtained an average value of α = 0.12 +/- 0.03 dB/um.

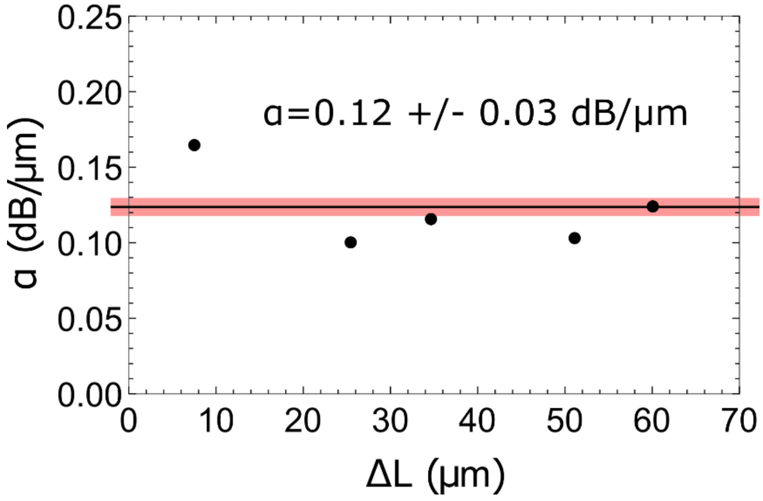

Figure S6. Propagation loss (α) as a function of the relative distance $\Delta L = (d_i - d_0)$ for each pair of measurements reported in Figure 2d of the main text.

Section 6: Grating efficiency and polariton nonlinearities

The laser was tuned to have a narrow momentum-space waist in order to have a precise control over the injection conditions and obtain a clean comparison between the ON and OFF states. Specifically, by adjusting the laser angle we achieve either efficient resonant injection of the polariton mode (ON state) or suppress injection by detuning the momentum (OFF state). The experimental configuration is illustrated in Figure S7a and S7b, where both the momentum-space and real-space distributions for the ON and OFF states are shown. The absolute grating coupling efficiency was then estimated by quantitatively analyzing the laser intensity corresponding to the ON and OFF states on the input grating. Figure S7c shows the integrated intensity (integrated over the area of the grating) for four different grating structures. The coupling efficiency was calculated using the relation $\varepsilon = (I_{OFF} - I_{ON}) / I_{OFF}$, yielding values in the range of 9–15%, depending on the specific structure.

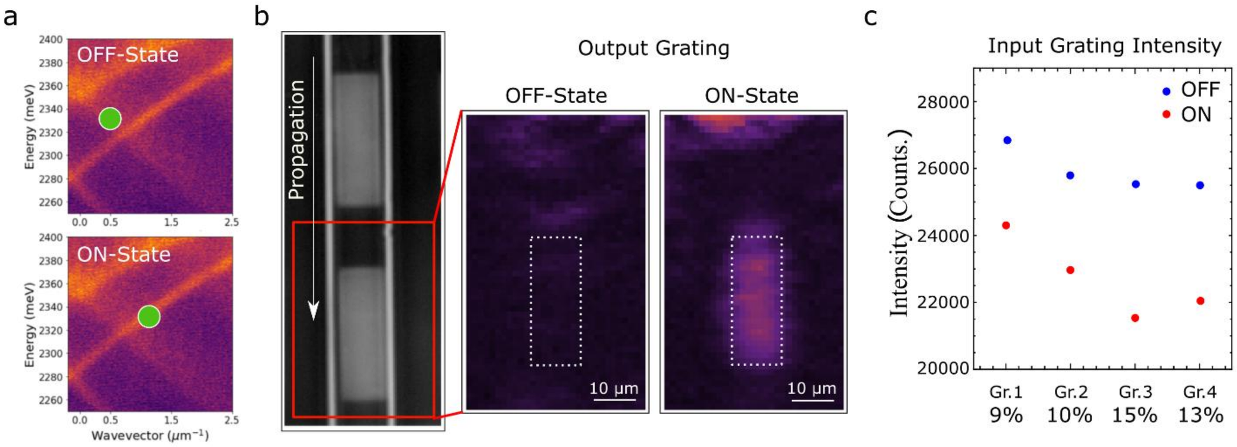

Figure S7 (a) Energy vs. in-plane momentum map illustrating the experimental configuration used to evaluate the grating coupling efficiency. A CW 532 nm laser (green circle) is positioned either off-resonance (top) or on-resonance (bottom) with the propagating polariton mode, at a given energy. (b) Corresponding real-space image on the output grating. (c) Laser intensity (integrated over the area of the grating) at the input grating for the off-resonance (blue dots) and on-resonance (red dots) conditions, shown for four different gratings (i.e., distinct perovskite wires). The extracted coupling efficiency ranges from 9% to 15% as indicated.

The nonlinear response under optical pumping in our samples is investigated by resonantly pumping one of the two gratings with a pulsed laser (10 kHz, 100 fs) in a reflection configuration (Figure S8). Zoomed-in views are shown for low-power excitation (Figure S8b) and high-power excitation (Figure S8c). The LP waveguide modes appear as dips in the laser spectrum, as observed in the corresponding reflection spectra obtained by taking a vertical slice

(integrated over 3 pixels) at $k \sim 0.7\ \mu m^{-1}$, collected at low excitation power (Figure S8d) and high excitation power (Figure S8e). An increase in pumping power leads to a blueshift of the LP branches.

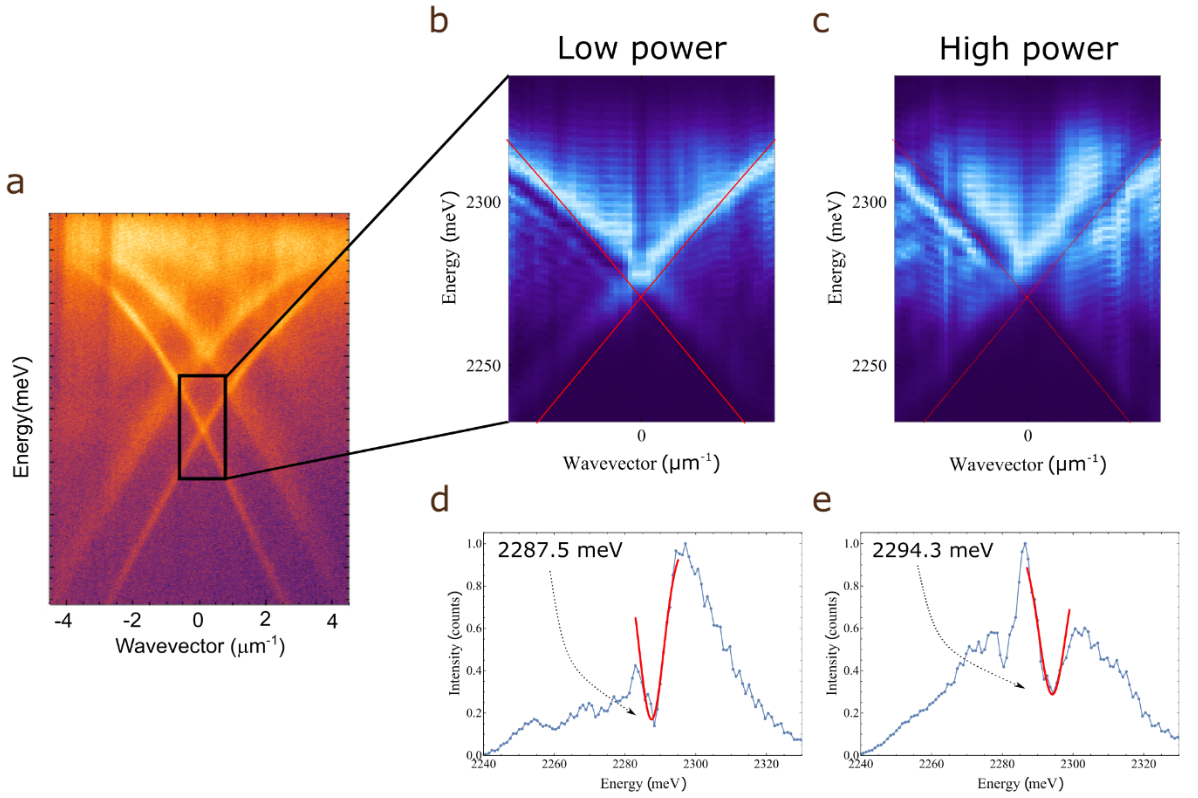

Figure S8. (a) Experimental energy versus in-plane momentum photoluminescence map. The white rectangle indicates the pumped area, with a zoomed-in view shown for low-power excitation (b) and high-power excitation (c). The corresponding reflection spectra obtained by taking a vertical slice (integrated over 3 pixels) at $k \sim 0.7\ \mu m^{-1}$ are displayed for the low-power regime (d) and the high-power regime (e).

Section 7: Key figures of merit of the switch

1) Propagation

In our perovskite-based waveguide system, we measured long-range polariton propagation exceeding 100 μm, limited only by the distance between the two gratings, and evaluated a loss coefficient α of approximately 0.12 dB/μm.

Regarding the use of perovskites, evidence of polariton propagation has been reported in planar microcavities. These include the propagation of a polariton condensate[2] and of a resonantly excited polariton wavepacket[3] . In both cases, $CsPbBr_3$ microwires were employed, and maximum propagation lengths of approximately 50–60 μm were observed. In one of our previous works, we also demonstrated polariton propagation in a 2D perovskite thick single crystal, where self-sustained exciton-polaritons exhibited propagation lengths of around 50–60 μm under resonant excitation[4]. More recently, a $MAPbBr_3$ perovskite waveguide film was realized, incorporating thermally imprinted in- and outcoupler gratings[5]. This system achieved propagation lengths between 50–200 μm.

Concerning the use of TMDs, polariton propagation lengths of approximately 10–20 μm have been reported in planar microcavities[6], as well as in thin waveguides[7], where a propagation length of about 12 μm was measured. Additionally, Bloch surface wave polariton systems incorporating TMDs have demonstrated longer propagation distances in the range of 30–60 μm[8,9].

For organic semiconductors, although they have been extensively studied, polariton propagation in planar microcavities is typically limited to just a few microns[10]. However, significantly longer propagation—ranging from 30 to 100 μm—has been demonstrated in Bloch surface wave polariton systems, enabled by the use of high-quality organic thin films[11].

Overall, the above discussed reports confirm the superior performance of waveguide-based polaritons compared to typical planar microcavities, which often suffer from limited propagation lengths and reduced flexibility for photonic integration. The recent demonstration of a $MAPbBr_3$ perovskite waveguide with thermally imprinted gratings is in good agreement with our observations, further supporting the potential of waveguide geometries for practical polaritonic devices. Moreover, our approach

benefits from the high crystallinity of the perovskite material, which contributes to reduced scattering losses and enhanced polariton transport.

2) Switching Contrast.

From the data reported in Figure 4, we evaluated the switching contrast of our device. Specifically, the switching contrast was calculated in decibels (dB) using the following relation: Switching Contrast (dB)=10Log[(I_{on}/I_{off})] where I_{on} and I_{off} represent the output intensities in the "on" and "off" states, respectively. Based on the data, we obtained a switching contrast between 8-11 dB, indicating a clear and robust switching behavior.

To compare this result with existing literature, we consider below polariton switches realized in different excitonic platforms operating at room temperature in experimental schemes close to the one employed here.

In the context of perovskites, an all-optical switch was implemented in a planar microcavity embedding $CsPbBr_3$ microwires[3]. In this work, the authors used a resonant beam to propagate within the microcavity plane and an additional beam to modulate the propagating signal at a different spatial position. The reported device exhibited a switching contrast of approximately 10 dB.

Similar implementations have also been investigated in TMD-based optical devices. A spin switch was recently demonstrated in a planar microcavity embedding WS_2 monolayers[12]. The implementation relies on the control of a quasi-resonant laser by introducing a weak control beam at a different in-plane momentum. By using a co-circular injection scheme, the system can be brought into the ON state (i.e., high transmission through the microcavity). When the polarization of the control beam is rotated to cross-circular polarization, while keeping the external power constant, the transmission is switched OFF. Under these experimental conditions, the system exhibits a switching contrast of approximately 8–10 dB between the co- and cross-circularly polarized excitation schemes.

Moreover, Bloch Surface Wave polaritons have been used to implement a nonlinear polariton source still employing a WS_2 monolayer[8]. The experiment is carried out by using a single beam slightly off resonance with the propagating polariton mode. By increasing the pumping power the beam gradually goes on resonance with the polariton branch, therefore injecting photons in a nonlinear way into the polariton mode. Based on the reported data, a switching contrast of around 6.5 dB can be retrieved between

the propagating polariton particles and the scattered signal. Last, exciton-polariton modes formed in an ultrathin WS_2 waveguide (spatially patterned to create a Mach-Zender interferometer) have been used to implement a switch[13]. By locally controlling one arm of the interferometer, a switching contrast of around 6 dB has been achieved. It is also worth mentioning that a polariton transistor[14] as well as OR and AND logic gates[15] have been successfully realized in organic planar microcavities. While these works represent important milestones, it is important to note that they rely on stimulation via vibronic replicas and condensate formation, which makes them fundamentally different from our experimental approach. As such, they do not offer a directly comparable basis for performance evaluation. The same is valid for other work on perovskite which relies on control and population switch of a polariton condensate[16].

3) Energy Threshold for Switching.

We calculate the incident energy of the pulsed laser as: $E_{tot} = P/R$, where P denotes the excitation power and R = 10 kHz is the repetition rate. In order to calculate the real injected energy into the system, we considered the attenuation of the excitation line (A_{line}) and the injection efficiency of the grating (ε = 9–15%, depending on the specific grating, as reported in Figure S9). Considering that the excitation spot area of the pulsed laser covers the entire input grating, we considered a pumping area of S ~ 260 μm^2 (the grating area), and calculate the energy threshold as $E_{inj} = E_{tot} * A_{line} * \varepsilon/S$. With reference to Figure 4 (ON state, with P around 1000 μW) we get a switching threshold in the range of 30-60 $\mu J/cm^2$.

It should be noted that a direct quantitative comparison of the switching energy threshold with other polariton devices realized in different excitonic materials is not straightforward. For instance, for the all-optical switch implemented in a planar microcavity embedding $CsPbBr_3$ microwires[3] the switching threshold is not directly reported. For the spin switch realized in a WS_2 microcavity[12] the power in reported in nW. For Bloch surface wave device a power of 600 W/cm^2 is reported as necessary to induce a blueshift of the polariton mode at 20% excitonic fraction. However, the switching experiment is conducted at different excitonic fraction (36%) requiring 5-10 uW to get in the ON-state. Last, the WS_2 Mach-Zender waveguide[13] employs an off-resonant CW laser with a pumping power of around 5 mW.

Section 8: Propagation loss mechanisms and strategies to improve coupling efficiency.

1) Roughness and intrinsic absorption

   The main contributions to propagation losses include scattering from sidewall roughness, material imperfections, and intrinsic absorption within the waveguide core. These effects lead to attenuation of light within the guided mode as it travels, reducing the intensity that reaches the output grating. Such losses are especially significant over long propagation distances. Future improvements could involve the optimization of the fabrication process—specifically, enhancing the quality of the silicon master used to create the polymeric replica that confines the perovskite precursors. Additionally, modifying the chemical composition of the PDMS (e.g., by using polymers with different atomic weights) could result in sharper, higher-fidelity replicas, thereby reducing sidewall roughness. It is also important to note that the switching experiments presented here using a 532 nm laser as propagating signal could be performed with a more detuned laser, well separated from the exciton absorption. Indeed, thanks to the large Rabi splitting, it is possible to retain a significant excitonic fraction even at energies well below the exciton resonance. This reduces intrinsic absorption while still enabling sufficient nonlinear interaction, thereby preserving switching functionality and improving overall performance.

2) Mode mismatch

   Another significant source of inefficiency arises from imperfect overlap between the excitation laser and the guided polariton mode at the input, as well as between the guided mode and the grating's out-coupling profile at the output. Even when momentum matching is carefully optimized, spatial mismatch can cause partial coupling into higher-order modes. These modes do not contribute effectively to guided propagation and are ultimately lost due to scattering or absorption, thereby reducing the overall device efficiency. Designing the waveguide to support a single mode could mitigate this issue by eliminating higher-order modes, improving mode overlap, and enhancing coupling efficiency and signal fidelity.

3) Grating efficiency

Although the employed strategy already enables effective coupling, losses at the input and output gratings can arise from suboptimal design parameters (e.g., period, duty cycle, etch depth) and fabrication imperfections. These factors reduce the coupling efficiency between free-space light and the guided polariton mode. Optimization of the grating bandwidth—which determines the range of energies and momenta over which efficient coupling occurs—could further enhance coupling performance. The use of apodized gratings—in which the grating is gradually modulated along its length—can also improve performance by minimizing back-reflection and enhancing mode matching, thereby optimizing both in- and out-coupling efficiency. Additionally, material compatibility during fabrication is critical. Among metals, only gold has good resistance to the precursors solution and is suitable for the proposed fabrication approach. However, gold gratings exhibit intrinsic absorption in the spectral region of interest and may also suffer undetectable damage or deformation during the growth process, compromising their performance. In this context, dielectric gratings are preferable due to their superior chemical and thermal stability as well as intrinsic minimal absorption, which helps to preserve the grating quality and improve the overall coupling efficiency.

References

(1) Fieramosca, A.; Mastria, R.; Dini, K.; Dominici, L.; Polimeno, L.; Pugliese, M.; Prontera, C. T.; De Marco, L.; Maiorano, V.; Todisco, F. Origin of Exciton–Polariton Interactions and Decoupled Dark States Dynamics in 2D Hybrid Perovskite Quantum Wells. *Nano Lett.* **2024**, *24* (27), 8240–8247.

(2) Su, R.; Wang, J.; Zhao, J.; Xing, J.; Zhao, W.; Diederichs, C.; Liew, T. C.; Xiong, Q. Room Temperature Long-Range Coherent Exciton Polariton Condensate Flow in Lead Halide Perovskites. *Sci. Adv.* **2018**, *4* (10), eaau0244.

(3) Feng, J.; Wang, J.; Fieramosca, A.; Bao, R.; Zhao, J.; Su, R.; Peng, Y.; Liew, T. C.; Sanvitto, D.; Xiong, Q. All-Optical Switching Based on Interacting Exciton Polaritons in Self-Assembled Perovskite Microwires. *Sci. Adv.* **2021**, *7* (46), eabj6627.

(4) Fieramosca, A.; De Marco, L.; Passoni, M.; Polimeno, L.; Rizzo, A.; Rosa, B. L.; Cruciani, G.; Dominici, L.; De Giorgi, M.; Gigli, G. Tunable Out-of-Plane Excitons in 2D Single-Crystal Perovskites. *Acs Photonics* **2018**, *5* (10), 4179–4185.

(5) Glebov, N. V.; Masharin, M. A.; Yulin, A.; Mikhin, A.; Miah, M. R.; Demir, H. V.; Krizhanovskii, D. N.; Kravtsov, V.; Samusev, A. K.; Makarov, S. V. Room-Temperature Exciton-Polariton-Driven Self-Phase Modulation in Planar Perovskite Waveguides. *ACS Nano* **2025**, *19* (14), 14097–14106.

(6) Wurdack, M.; Estrecho, E.; Todd, S.; Yun, T.; Pieczarka, M.; Earl, S. K.; Davis, J. A.; Schneider, C.; Truscott, A. G.; Ostrovskaya, E. A. Motional Narrowing, Ballistic Transport, and Trapping of Room-Temperature Exciton Polaritons in an Atomically-Thin Semiconductor. *Nat. Commun.* **2021**, *12* (1), 5366.

(7) Hu, F.; Luan, Y.; Scott, M. E.; Yan, J.; Mandrus, D. G.; Xu, X.; Fei, Z. Imaging Exciton–Polariton Transport in MoSe2 Waveguides. *Nat. Photonics* **2017**, *11* (6), 356–360.

(8) Barachati, F.; Fieramosca, A.; Hafezian, S.; Gu, J.; Chakraborty, B.; Ballarini, D.; Martinu, L.; Menon, V.; Sanvitto, D.; Kéna-Cohen, S. Interacting Polariton Fluids in a Monolayer of Tungsten Disulfide. *Nat. Nanotechnol.* **2018**, *13* (10), 906–909.

(9) Liu, B.; Lynch, J.; Zhao, H.; Conran, B. R.; McAleese, C.; Jariwala, D.; Forrest, S. R. Long-Range Propagation of Exciton-Polaritons in Large-Area 2D Semiconductor Monolayers. *ACS Nano* **2023**, *17* (15), 14442–14448.

(10) Lerario, G.; Fieramosca, A.; Barachati, F.; Ballarini, D.; Daskalakis, K. S.; Dominici, L.; De Giorgi, M.; Maier, S. A.; Gigli, G.; Kéna-Cohen, S. Room-Temperature Superfluidity in a Polariton Condensate. *Nat. Phys.* **2017**, *13* (9), 837–841.

(11) Lerario, G.; Ballarini, D.; Fieramosca, A.; Cannavale, A.; Genco, A.; Mangione, F.; Gambino, S.; Dominici, L.; De Giorgi, M.; Gigli, G. High-Speed Flow of Interacting Organic Polaritons. *Light Sci. Appl.* **2017**, *6* (2), e16212–e16212.

(12) Zhao, J.; Fieramosca, A.; Bao, R.; Dini, K.; Su, R.; Sanvitto, D.; Xiong, Q.; Liew, T. C. Room Temperature Polariton Spin Switches Based on Van Der Waals Superlattices. *Nat. Commun.* **2024**, *15* (1), 7601.

(13) Lee, S. W.; Lee, J. S.; Choi, W. H.; Choi, D.; Gong, S.-H. Ultra-Compact Exciton Polariton Modulator Based on van Der Waals Semiconductors. *Nat. Commun.* **2024**, *15* (1), 2331.

(14) Zasedatelev, A. V.; Baranikov, A. V.; Urbonas, D.; Scafirimuto, F.; Scherf, U.; Stöferle, T.; Mahrt, R. F.; Lagoudakis, P. G. A Room-Temperature Organic Polariton Transistor. *Nat. Photonics* **2019**, *13* (6), 378–383.

(15) Zasedatelev, A. V.; Baranikov, A. V.; Sannikov, D.; Urbonas, D.; Scafirimuto, F.; Shishkov, V. Y.; Andrianov, E. S.; Lozovik, Y. E.; Scherf, U.; Stöferle, T. Single-Photon Nonlinearity at Room Temperature. *Nature* **2021**, *597* (7877), 493–497.

(16) Masharin, M. A.; Oskolkova, T.; Isik, F.; Volkan Demir, H.; Samusev, A. K.; Makarov, S. V. Giant Ultrafast All-Optical Modulation Based on Exceptional Points in Exciton–Polariton Perovskite Metasurfaces. *ACS Nano* **2024**, *18* (4), 3447–3455.
